# Sustainable Wheat Cultivation in Sandy Soils: Impact of Organic and Biofertilizer Use on Soil Health and Crop Yield

**DOI:** 10.3390/plants13223156

**Published:** 2024-11-10

**Authors:** Ibrahim El-Akhdar, Mahmoud M. A. Shabana, Nagwa M. M. El-Khateeb, Nevien Elhawat, Tarek Alshaal

**Affiliations:** 1 Department of Microbiology, Soils, Water and Environment Research Institute, Agriculture Research Center (ARC), Giza 12112, Egypt; dr.elakhdar@yahoo.com; 2 Soils, Water and Environment Research Institute, Agricultural Research Center (ARC), Giza 12112, Egypt; mahmoud.shabana@arc.sci.eg; 3 Agricultural Microbiology, Agricultural Botany Department, Faculty of Agriculture, Kafrelsheikh University, Kafr El-Sheikh 33516, Egypt; nagwa.elkhateeb@yahoo.com; 4 Department of Applied Plant Biology, Faculty of Agricultural and Food Sciences and Environmental Management, University of Debrecen, Böszörményi Str. 138, 4032 Debrecen, Hungary; 5 Faculty of Agriculture (for Girls), Al-Azhar University, Cairo 11884, Egypt; 6 Soil and Water Science Department, Faculty of Agriculture, Kafrelsheikh University, Kafr El-Sheikh 33516, Egypt

**Keywords:** organic fertilizers, soil health, soil organic matter, macronutrients, microbial activity

## Abstract

Sandy soils are widespread globally and are increasingly utilized to meet the demands of a growing population and urbanization for food, fiber, energy, and other essential services. However, their poor water and nutrient retention makes crop cultivation challenging. This study evaluated the effects of integrating compost and plant growth-promoting rhizobacteria (PGPR; *Azospirillum brasilense* SWERI 111 and *Azotobacter chroococcum* OR512393) on wheat (*Triticum aestivum* L. var. Misr 1) grown in sandy soil under varying levels of recommended NPK (50%, 75%, and 100%) fertilization. Conducted over two growing seasons, the experiment aimed to assess soil health, nutrient uptake, microbial activity, and plant productivity in response to compost and PGPR treatments. The results demonstrated that combining compost and PGPR significantly improved soil chemical properties, such as reducing soil pH, electrical conductivity (ECe), and sodium adsorption ratio (SAR), while enhancing soil organic matter (SOM). Additionally, compost and PGPR improved soil nutrient content (N, P, K) and boosted the total bacterial and fungal counts. The combined treatment also increased urease and phosphatase enzyme activities, contributing to enhanced nutrient availability. Notably, plant productivity was enhanced with compost and PGPR, reflected by increased chlorophyll and reduced proline content, along with improved grain and straw yields. Overall, the results underscore the potential of compost and PGPR as effective, sustainable soil amendments to support wheat growth under varying NPK levels.

## 1. Introduction

The global population, projected to rise from 7.8 billion in 2020 to 9 billion by 2050 [1], underscores the urgent need for extensive measures to address food security and nutrition. These efforts must target the entire food value chain, including food production [2]. One proposed solution to food insecurity is sustainable agricultural intensification, which involves diversifying crops, adopting more efficient production methods, and implementing strategies to mitigate climate change [3]. However, vast areas of land worldwide, due to their low productivity and high permeability to water and nutrients, are unsuitable for traditional farming. Among these, sandy soils, which cover approximately 900 million hectares globally [4], pose a particular challenge. Their high permeability not only limits crop yields [5] but also increases the risk of groundwater contamination from fertilizers [6], highlighting the need for technological interventions.

In arid and semi-arid regions where sandy soils are widespread, the anticipated effects of climate change and variability are expected to worsen food security and nutrition issues for farmers who depend on these lands [7]. To improve crop yields and ensure food security, technologies that reduce water and nutrient loss from sandy soils are crucial. Several techniques have been developed to enhance the moisture and nutrient retention of these soils. These include water retention technologies designed to maximize rainwater use and reduce irrigation needs around plant roots [8]. For instance, biochar application has been shown to improve the hydro-physical properties of sandy soil, aiding water conservation in arid and semi-arid environments [9]. Other methods, such as using asphalt to create a barrier under the soil to limit water movement, and soil management techniques like mulching and cover cropping, help retain moisture [10,11]. Additionally, organic amendments, including compost, food waste, crop residues, and manure, have been found to boost sandy soil productivity by increasing organic matter, enhancing microbial diversity, and improving moisture and nutrient retention [12].

Sandy soils in Egypt represent more than 70% of total area. Most of these soils may be reclaimed with low costs compared with other desert soils. Also, these soils are more suitable to much economical cultivation such as wheat, barley and corn. In addition, such soils are located within or near to the Valley of Nile River [4]. In fragile ecosystems where effective water and nutrient management are essential, relying on conventional chemical fertilizers may not offer sustainable long-term benefits. Although these fertilizers can quickly supply nutrients, excessive use can degrade the soil, cause pollution, and further harm soil health. To overcome these challenges, sustainable soil management techniques, including the use of organic fertilizers and biofertilizers, have emerged as promising solutions to enhance the fertility and productivity of sandy soils [13].

Organic fertilizers, sourced from plant and animal materials, provide essential nutrients and help improve soil structure and organic matter over time. By enhancing the water retention and nutrient availability of sandy soils, these organic amendments promote healthier plant growth while reducing dependence on synthetic inputs [14]. In contrast, biofertilizers introduce beneficial microorganisms, such as plant growth-promoting rhizobacteria (PGPR), into the soil. These microorganisms increase nutrient availability by fixing atmospheric nitrogen, solubilizing phosphates, and producing growth-enhancing compounds. The combined use of organic fertilizers and biofertilizers offers a sustainable, environmentally friendly approach to restoring and maintaining soil health [15].

The application of compost and PGPR has shown promising benefits for wheat cultivation, particularly in nutrient-deficient and low-water-holding soils like sandy soils. Compost enriches soil organic matter, which improves its structure, nutrient-holding capacity, and water retention, essential for supporting wheat growth under low-fertility conditions [16]. Additionally, compost supplies slow-release nutrients that enhance wheat productivity while reducing dependence on chemical fertilizers, a sustainable approach in sandy soils where nutrient leaching is prevalent [17]. On the other hand, PGPR, including strains like *Azospirillum brasilense* and *Azotobacter chroococcum*, contribute significantly to nutrient availability by fixing atmospheric nitrogen and solubilizing phosphorus, both crucial for wheat growth [15]. PGPR also enhance wheat root growth and increase the plants’ resilience to environmental stresses such as salinity and drought by stimulating stress-related compounds, which is especially advantageous in sandy soils prone to moisture loss [1]. Studies combining compost and PGPR in wheat production have reported synergistic effects, where compost creates a conducive environment for PGPR to thrive, further amplifying microbial activity and nutrient cycling that lead to better plant growth and yield [11]. This integrated approach thus represents a sustainable strategy to improve wheat productivity and soil health in challenging agricultural settings.

Sandy soils, characterized by high permeability and low nutrient retention, pose significant challenges for sustainable agriculture, often leading to reliance on chemical fertilizers. While these fertilizers provide rapid nutrient delivery, excessive use degrades soil health, contaminates groundwater, and harms soil structure [18]. Organic amendments like compost have been shown to improve soil organic matter and nutrient retention, though they typically require long-term application for substantial benefits [19]. Additionally, biofertilizers, such as PGPR, enhance nutrient uptake and microbial activity in the root zone, but their efficacy depends on favorable soil conditions, particularly in nutrient-poor, low-organic-matter sandy soils [15]. The current study addresses these gaps by investigating the combined use of compost and PGPR, hypothesizing that this integrated approach can more effectively sustain soil fertility and enhance wheat productivity in sandy soils. Compost provides organic matter that increases nutrient retention and soil moisture, while PGPR aids nutrient availability by fixing nitrogen and solubilizing phosphorus. This combination not only enhances microbial diversity and activity but also promotes nutrient cycling and organic matter decomposition, which are crucial for soil fertility in sandy environments [20]. Furthermore, compost contributes to water retention, and the root-promoting effects of PGPR allow plants to access nutrients even under limited water conditions. By testing this integrated method under reduced NPK levels, the study demonstrates a sustainable approach to support high-yield wheat production and long-term soil health in challenging sandy soils. This research thus highlights the potential of combining organic and microbial amendments to mitigate the limitations of sandy soils and reduce dependency on chemical inputs.

## 2. Results

### 2.1. Alterations in Soil Chemical Properties

**Figure 1:**
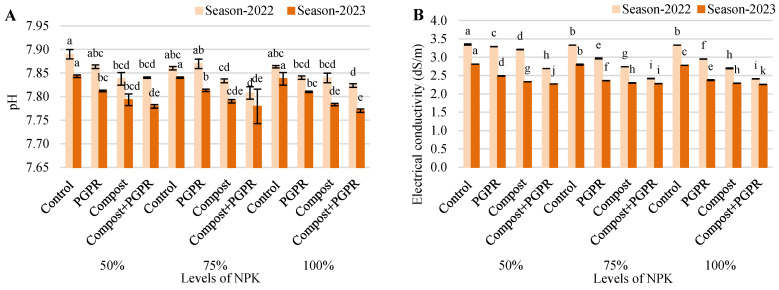

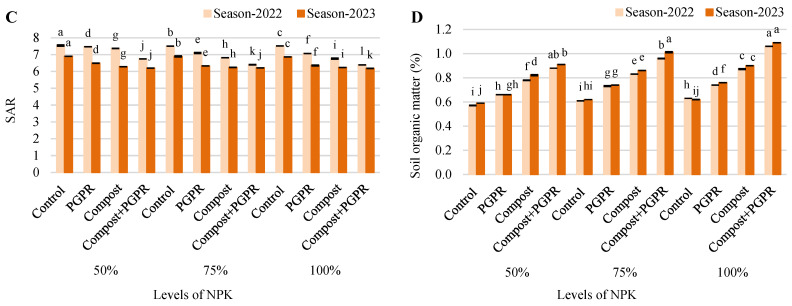
Responses of soil traits: (**A**) pH, (**B**) electrical conductivity, (**C**) soil organic matter, and (**D**) sodium adsorption ratio after treating wheat plants (*Triticumae stivum* L.) grown in sand soil and irrigated with low-quality water with compost, plant growth-promoting rhizobacteria (PGPR), and their combination under three levels of recommended NPK (50, 75 and 100%) during two seasons (2022 and 2023). Different letters above bars of the same season are significant at the level of *p* ≤ 0.05 according to the LSD test.

The pH results from the experiment reveal minor variations across the 2022 and 2023 growing seasons, indicating that different treatments had a minimal impact on soil pH levels (Figure 1A). In the 50% NPK treatment, the control group showed a slight decrease in pH from 7.89 in 2022 to 7.84 in 2023. The PGPR treatment followed a similar trend, with a reduction from 7.86 to 7.81, while the compost treatment saw a decrease from 7.84 to 7.79. The compost + PGPR combination resulted in a pH decline from 7.84 to 7.78. In the 75% NPK treatment, the control group remained stable at 7.86 in 2022 and slightly dropped to 7.84 in 2023. The PGPR treatment decreased from 7.87 to 7.81, and the compost treatment saw a reduction from 7.83 to 7.79, while the compost + PGPR combination showed a slight drop from 7.81 to 7.78. In the 100% NPK treatment, the control group remained steady at 7.86 in 2022 and decreased to 7.84 in 2023. The PGPR treatment reduced from 7.84 to 7.81, while the compost treatment dropped from 7.84 to 7.78. The compost + PGPR combination resulted in the lowest pH values, with a decrease from 7.82 to 7.77. Overall, the data suggest that compost and PGPR treatments had minimal but consistent effects on lowering soil pH, which remained within a neutral to slightly alkaline range across both growing seasons.

The results for EC_e_ across the 2022 and 2023 growing seasons demonstrate a notable decrease in soil EC_e_ levels over time for all treatments (Figure 1B). In the 50% NPK treatment, the control group saw a drop from 3.35 dS/m in 2022 to 2.81 dS/m in 2023. The PGPR treatment showed a more pronounced decline from 3.29 dS/m to 2.49 dS/m, while the compost treatment reduced EC from 3.21 dS/m to 2.33 dS/m. The combination of compost and PGPR resulted in the most significant decrease, from 2.69 dS/m to 2.27 dS/m. In the 75% NPK treatment, the control group’s EC_e_ fell from 3.33 dS/m to 2.80 dS/m, with the PGPR treatment reducing EC_e_ from 2.97 dS/m to 2.36 dS/m. The compost treatment lowered EC_e_ from 2.74 dS/m to 2.30 dS/m, while the compost + PGPR treatment showed a more modest decline from 2.42 dS/m to 2.28 dS/m. For the 100% NPK treatment, the control group’s EC_e_ decreased from 3.33 dS/m to 2.78 dS/m, while the PGPR treatment dropped from 2.95 dS/m to 2.38 dS/m. The compost treatment reduced EC_e_ from 2.70 dS/m to 2.29 dS/m, and the compost + PGPR combination recorded the lowest EC_e_ values, dropping from 2.41 dS/m to 2.26 dS/m. Overall, the data indicate that compost and PGPR treatments consistently reduce soil EC_e_ levels across both growing seasons, with the combined treatment being the most effective in maintaining lower conductivity values.

The SAR data from the experiment across the 2022 and 2023 seasons show a clear downward trend in soil SAR values, with compost and PGPR treatments leading to notable reductions (Figure 1C). In the 50% NPK treatment, the control group experienced a decrease in SAR from 7.54 in 2022 to 6.90 in 2023. The PGPR treatment reduced SAR more significantly, from 7.47 to 6.49, while compost treatment dropped it from 7.37 to 6.28. The combination of compost and PGPR was the most effective, lowering SAR from 6.75 to 6.20. In the 75% NPK treatment, the control group saw a decrease from 7.51 to 6.89, while the PGPR treatment reduced SAR from 7.10 to 6.32. The compost treatment showed a decline from 6.81 to 6.24, and the compost + PGPR treatment resulted in the lowest SAR values, dropping from 6.40 to 6.21. In the 100% NPK treatment, the control group decreased from 7.51 to 6.86, the PGPR treatment from 7.07 to 6.35, and the compost treatment from 6.77 to 6.23. The compost + PGPR combination had the lowest SAR values in this group, falling from 6.39 to 6.18. Overall, the compost and PGPR treatments consistently lowered SAR levels across both growing seasons, with the compost + PGPR combination proving to be the most effective in reducing sodium buildup in the soil.

The data of SOM content across the 2022 and 2023 growing seasons highlight the positive effects of compost and PGPR treatments on soil fertility (Figure 1D). In the 50% NPK treatment, the control group showed a slight increase in SOM from 0.57% to 0.59%. PGPR treatments maintained a stable SOM content of 0.66% in both years, while compost alone improved SOM from 0.78% to 0.82%. The combination of compost and PGPR resulted in the highest increase, from 0.88% to 0.91%. For the 75% NPK treatment, the control group exhibited a small rise from 0.61% to 0.62%, and the PGPR treatment increased from 0.73% to 0.74%. Compost alone increased SOM from 0.83% to 0.86%, and the compost + PGPR combination produced the highest gains, from 0.96% to 1.01%. In the 100% NPK group, the control maintained SOM around 0.62–0.63%, while the PGPR treatment led to a slight increase from 0.74% to 0.76%. Compost alone boosted SOM from 0.87% to 0.90%, and the compost + PGPR treatment saw the largest increase, from 1.06% to 1.09%. These results indicate that compost and PGPR treatments, especially in combination, consistently enhanced organic matter content in the soil across both seasons, contributing to improved soil health and fertility.

### 2.2. Changes in Soil NPK Contents

**Figure 2:**
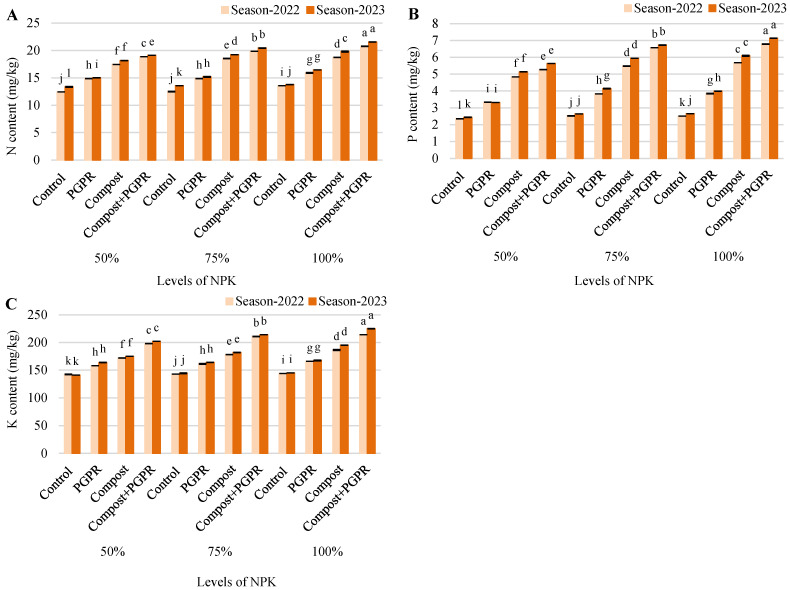
Responses of soil NPK: (**A**) N, (**B**) P, and (**C**) K, after treating wheat plants (*Triticumae stivum* L.) grown in sand soil and irrigated with low-quality water with compost, plant growth-promoting rhizobacteria (PGPR), and their combination under three levels of recommended NPK (50, 75, and 100%) during two seasons (2022 and 2023). Different letters above bars of the same season are significant at the level of *p* ≤ 0.05 according to the LSD test.

The N content data for the 2022 and 2023 seasons illustrate the substantial impact of different treatments, particularly the combination of compost and PGPR (Figure 2A). In the 50% NPK treatment, the control group showed an increase in N from 12.4 to 13.3. PGPR treatment resulted in a more significant rise, from 14.9 to 15.0, while compost application saw an even greater boost, from 17.5 to 18.2. The combination of compost and PGPR yielded the highest nitrogen levels, increasing from 18.9 to 19.1. For the 75% NPK treatment, the control increased from 12.5 to 13.6, while PGPR rose from 14.9 to 15.2. Compost alone raised N levels from 18.5 to 19.2, and compost + PGPR resulted in the highest increase, from 19.9 to 20.4. In the 100% NPK treatment, the control group showed a slight increase from 13.6 to 13.8, and the PGPR treatment raised N levels from 15.9 to 16.5. Compost application significantly boosted nitrogen from 18.7 to 19.8, and the compost + PGPR combination resulted in the highest N content, increasing from 20.8 to 21.5. These results demonstrate the effectiveness of compost and PGPR, particularly when combined, in enhancing nitrogen levels across different NPK rates and seasons, leading to improved soil fertility and plant productivity.

The P content results for the 2022 and 2023 seasons demonstrate the influence of different treatments on soil P levels (Figure 2B). In the 50% NPK treatment, the control group saw a modest increase in P, from 2.35 to 2.44. The PGPR treatment led to a P increase from 3.34 to 3.32, while compost application raised it significantly from 4.84 to 5.14. The combination of compost and PGPR yielded the highest P content, increasing from 5.27 to 5.63. In the 75% NPK treatment, the control group saw an increase from 2.53 to 2.64, while PGPR treatment increased P levels from 3.83 to 4.14. Compost application led to a larger rise, from 5.48 to 5.94, and the compost + PGPR combination again yielded the highest P levels, increasing from 6.57 to 6.73. In the 100% NPK treatment, the control group showed an increase from 2.51 to 2.65, with PGPR treatment raising P from 3.85 to 3.99. Compost increased P from 5.68 to 6.10, and the highest P levels were observed with compost + PGPR, which raised P from 6.79 to 7.13. Overall, the combination of compost and PGPR consistently provided the greatest boost to phosphorus content, highlighting their synergistic effect on improving soil fertility.

The K content data for the 2022 and 2023 seasons illustrate the varying impact of different treatments on soil K levels (Figure 2C). In the 50% NPK treatment, the control group showed a slight decrease in K, from 142 to 141, while the PGPR treatment increased K from 158 to 164. Compost application raised K levels further, from 172 to 175, with the compost + PGPR combination delivering the highest increase, from 198 to 202. In the 75% NPK treatment, the control group saw a slight rise, from 143 to 144, with the PGPR treatment increasing K from 161 to 164. Compost raised K levels from 178 to 182, and the compost + PGPR combination boosted it significantly from 211 to 214. In the 100% NPK treatment, the control group had a small rise, from 144 to 145, while PGPR raised K from 166 to 167. Compost application caused a notable increase, from 186 to 195, and the compost + PGPR combination again delivered the highest rise, from 214 to 225. Overall, the combination of compost and PGPR consistently resulted in the greatest improvement in potassium content across all treatments, emphasizing the effectiveness of this combination in enhancing soil K levels.

### 2.3. Changes in Total Counts of Bacteria and Fungi

**Figure 3:**
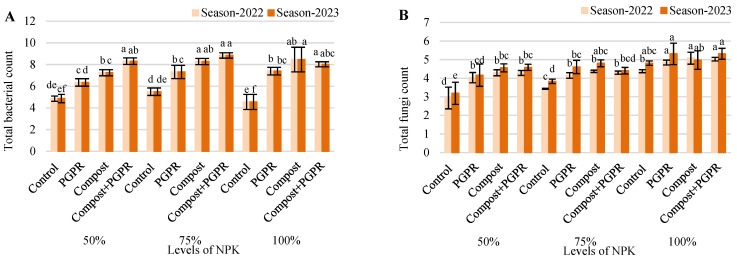
Alterations in (**A**) total bacterial count (×10^5^ CFU/g dry soil) and (**B**) total fungi count (×10^3^ CFU/g dry soil) after treating wheat plants (*Triticumae stivum* L.) grown in sand soil and irrigated with low-quality water with compost, plant growth-promoting rhizobacteria (PGPR), and their combination under three levels of recommended NPK (50, 75, and 100%) during two seasons (2022 and 2023). Different letters above bars of the same season are significant at the level of *p* ≤ 0.05 according to the LSD test.

The total bacterial count (TBC) results for the 2022 and 2023 seasons highlight the influence of various treatments on microbial activity in the soil (Figure 3A). In the 50% NPK treatment, the control group showed a stable TBC of 4.85 in 2022 and 4.86 in 2023. The PGPR treatment significantly increased TBC to 6.35 in both seasons, while the application of compost alone raised TBC further to 7.24. The combination of compost and PGPR delivered the highest TBC, reaching 8.31. In the 75% NPK treatment, the control group had a TBC of 5.50 across both years, while PGPR treatment increased this to 7.32. Compost application further raised TBC to 8.28, with the compost + PGPR combination resulting in the highest TBC of 8.84. In the 100% NPK treatment, the control group had a lower TBC of 4.55, but PGPR treatment raised it significantly to 7.39, with compost application increasing it further to 8.46. Interestingly, the combination of compost and PGPR in this case showed a slightly lower TBC of 8.03. Overall, the data indicate that compost and PGPR, either individually or combined, substantially enhance microbial activity, with the compost + PGPR combination generally achieving the highest TBC across most treatments.

The total fungal count (TFC) results from the 2022 and 2023 growing seasons reflect how different treatments impact fungal populations in the soil (Figure 3B). In the 50% NPK treatment, the control group exhibited a slight increase in TFC from 2.94 in 2022 to 3.19 in 2023. The PGPR treatment significantly boosted TFC to 4.03 in 2022 and 4.16 in 2023, while compost alone further elevated TFC to 4.29 in 2022 and 4.56 in 2023. The combination of compost and PGPR yielded the highest TFC, at 4.59 in 2023. In the 75% NPK treatment, the control group’s TFC increased from 3.43 to 3.83 between the two seasons, with the PGPR treatment pushing it higher to 4.15 in 2022 and 4.61 in 2023. The application of compost alone reached a TFC of 4.81 in 2023, but the combination of compost + PGPR remained consistent with lower values at 4.30 in 2022 and 4.41 in 2023. For the 100% NPK treatment, the control group had the highest baseline TFC, reaching 4.81 in 2023, while the PGPR treatment elevated this to 5.31 in the same year. Compost alone slightly reduced TFC in 2023 compared to 2022, from 5.08 to 4.98. Interestingly, the compost + PGPR combination, despite maintaining high levels, fluctuated with values of 5.03 in 2022 and 5.32 in 2023. In conclusion, PGPR and compost, whether applied individually or in combination, generally led to increases in TFC across all NPK treatments. The highest fungal activity was observed in the 100% NPK treatment, particularly when compost and PGPR were used together.

### 2.4. Responses of Phosphate-Solubilizing Bacteria and Soil Enzymes Activity

**Figure 4:**
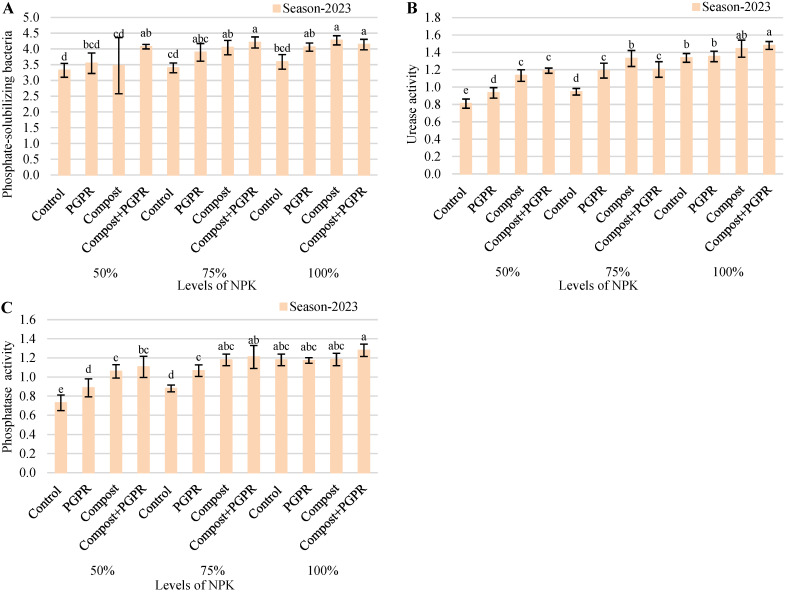
Variations in (**A**) phosphate-solubilizing bacteria (×10^3^ CFU/ g dry soil), (**B**) urease activity (mg NH_4_^+^/g soil/h), and (**C**) phosphatase activity (µmol p-nitrophenol/g soil/h) after treating wheat plants (*Triticumae stivum* L.) grown in sand soil and irrigated with low-quality water with compost, plant growth-promoting rhizobacteria (PGPR), and their combination under three levels of recommended NPK (50, 75, and 100%) during two seasons (2022 and 2023). Different letters above bars of the same season are significant at the level of *p* ≤ 0.05 according to the LSD test.

The analysis of biochemical parameters, including phosphate-solubilizing (P-solubilizing) bacteria, urease (mg NH_4_^+^/g soil/h), and phosphatase (µmol p-nitrophenol/g soil/h) activities for the 2023 growing season, demonstrates the effects of various treatments on plant productivity (Figure 4). In the 50% NPK treatment, the control group exhibited a P-solubilizing value of 3.32, while treatments with PGPR, compost, and the combination of compost + PGPR showed increased values of 3.55, 3.47, and 4.07, respectively. The urease activity increased from 0.81 in the control to 0.93 with PGPR, 1.13 with compost, and peaked at 1.19 with compost + PGPR. Similarly, phosphatase activity also improved, rising from 0.73 in the control to 0.89 in PGPR, 1.06 in compost, and 1.11 in the compost + PGPR treatment. For the 75% NPK treatment, the control group had P-solubilizing values of 3.40, which increased significantly with PGPR (3.89), compost (4.04), and the compost + PGPR combination (4.21). Urease activity showed a similar trend, from 0.95 in the control to 1.19 with PGPR, 1.33 with compost, and 1.20 with compost + PGPR. The phosphatase activity also increased, reaching values of 0.88, 1.07, 1.18, and 1.21 across the treatments, respectively.

In the 100% NPK treatment, the control group’s P-solubilizing activity was recorded at 3.59, while PGPR, compost, and compost + PGPR treatments showed higher values of 4.06, 4.27, and 4.14, respectively. Urease activity in the control group was at 1.34, increasing to 1.35 with PGPR, 1.44 with compost, and peaking at 1.48 with compost + PGPR. Phosphatase activity remained consistent, ranging from 1.18 in the control and PGPR treatments to 1.28 with compost + PGPR. Overall, these results indicate that the application of PGPR and compost, both individually and in combination, significantly enhances P-solubilizing activity, urease, and phosphatase levels in plants, suggesting improved nutrient availability and stress response under varying NPK treatments.

### 2.5. Changes in Chlorophyll and Proline Contents in Wheat Plants

**Figure 5:**
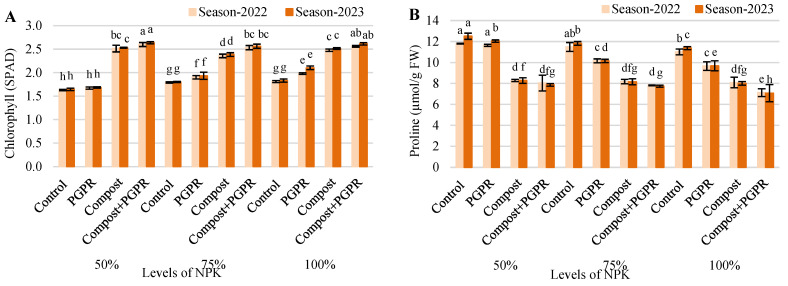
Variations in (**A**) relative chlorophyll content (SPAD) and (**B**) proline content (µmol/g FW), after treating wheat plants (*Triticumae stivum* L.) grown in sand soil and irrigated with low-quality water with compost, plant growth-promoting rhizobacteria (PGPR) and their combination under three levels of recommended NPK (50, 75, and 100%) during two seasons (2022 and 2023). Different letters above bars of the same season are significant at the level of *p* ≤ 0.05 according to the LSD test.

The results of the research experiment demonstrate the significant impact of different treatments on relative chlorophyll levels (SPAD) in plants across the 2022 and 2023 growing seasons (Figure 5). Overall, combinations of N, P, and K with compost and PGPR enhanced chlorophyll content compared to the control groups (Figure 5A). In the 50% NPK treatment, the chlorophyll levels for the control, PGPR, compost, and compost + PGPR treatments were 1.63, 1.67, 2.51, and 2.60, respectively, in 2022, and showed slight increases in 2023, with values of 1.64, 1.69, 2.53, and 2.64. The 75% NPK treatment revealed similar trends, with control (1.79 to 1.80), PGPR (1.90 to 1.93), compost (2.36 to 2.38), and compost + PGPR (2.53 to 2.56). Notably, in the 100% NPK treatment, the control group showed chlorophyll levels of 1.81 in 2022 and 1.83 in 2023, while PGPR treatments resulted in 1.98 and 2.10, compost treatments yielded 2.48 and 2.51, and compost + PGPR showed 2.56 and 2.61. These findings indicate that integrating compost and PGPR with NPK treatments positively influences plant chlorophyll levels, contributing to improved plant productivity across seasons.

The data from the research experiment illustrate the variations in proline content (µmol/g FW) among different treatments across the 2022 and 2023 growing seasons (Figure 5B). In the 50% NPK treatment, the control group had proline levels of 11.80 µmol/g in 2022 and increased to 12.51 µmol/g in 2023. The PGPR treatment yielded slightly lower proline levels at 11.63 in 2022 and decreased to 12.05 in 2023, while compost treatments showed lower values of 8.29 for both years, and compost + PGPR recorded the lowest levels at 8.05 and 7.87, respectively. In the 75% NPK treatment, control showed a slight increase from 11.48 µmol/g in 2022 to 11.83 µmol/g in 2023, while PGPR decreased from 10.16 to 10.15 µmol/g. Compost treatments remained stable at 8.19 and 8.17, while compost + PGPR showed a decrease from 7.82 to 7.75 µmol/g. For the 100% NPK treatment, control groups recorded 11.01 µmol/g in 2022 and increased to 11.37 µmol/g in 2023, while PGPR levels decreased from 9.67 to 9.69 µmol/g. Compost and compost + PGPR treatments had consistently low proline levels, ranging from 8.09 to 8.01 and 7.13 to 7.08, respectively. These findings indicate that while control and PGPR treatments generally resulted in higher proline levels, the application of compost and compost + PGPR was associated with reduced proline content, suggesting differences in stress responses and potential impacts on plant productivity across the two growing seasons.

### 2.6. Changes in NPK Contents in Grain and Straw

**Figure 6:**
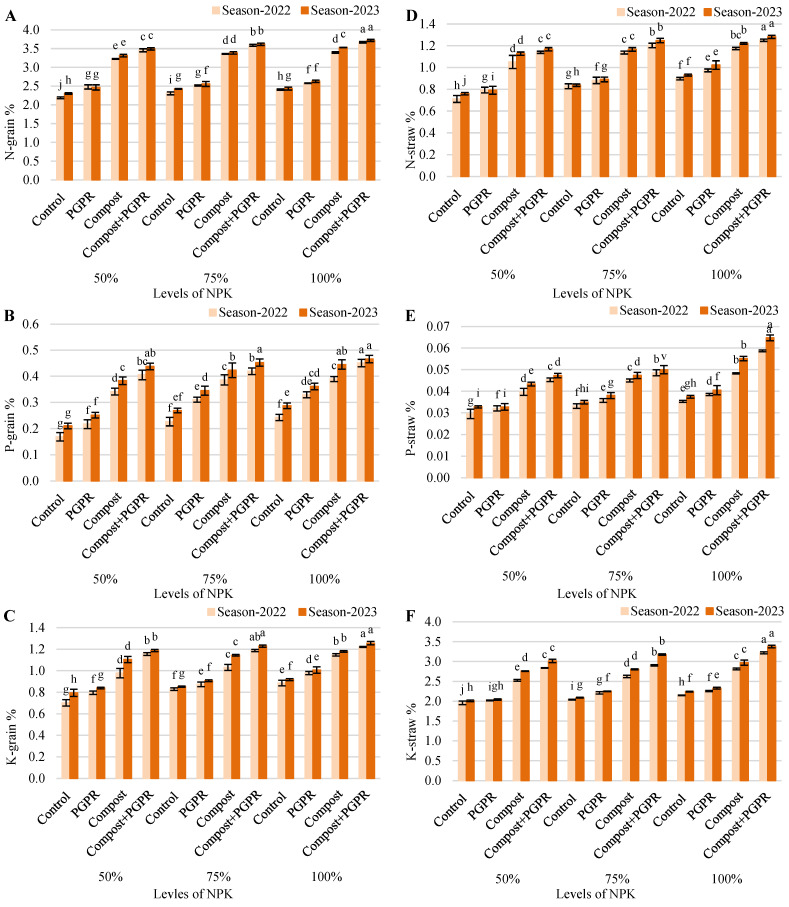
Variations in (**A**) grain N, (**B**) grain P, (**C**) grain K, (**D**) straw N, (**E**) straw P, and (**F**) straw K contents after treating wheat plants (*Triticumae stivum* L.) grown in sand soil and irrigated with low-quality water with compost, plant growth-promoting rhizobacteria (PGPR), and their combination under three levels of recommended NPK (50, 75, and 100%) during two seasons (2022 and 2023). Different letters above bars of the same season are significant at the level of *p* ≤ 0.05 according to the LSD test.

The results from the research experiment reveal significant differences in grain N content (%), showcasing the effectiveness of various treatments across the 2022 and 2023 growing seasons (Figure 6A). In the 50% NPK treatment, the control group exhibited grain N levels of 2.19% in 2022, increasing to 2.30% in 2023. The PGPR treatment had higher levels at 2.48% in 2022, slightly decreasing to 2.47% in 2023. The compost treatment recorded an increase from 3.22% to 3.31%, while the compost + PGPR combination yielded the highest levels, rising from 3.45% to 3.49%. The 75% NPK treatment followed a similar trend, with control values at 2.31% and 2.43%, PGPR at 2.52% and 2.56%, compost at 3.36% and 3.39%, and compost + PGPR showing the most significant increase from 3.59% to 3.62%. In the 100% NPK treatment, the control group recorded 2.41% in 2022 and 2.44% in 2023. PGPR levels were 2.58% in 2022 and increased to 2.63% in 2023. Compost treatments showed an increase from 3.40% to 3.53%, while compost + PGPR resulted in the highest values, rising from 3.67% to 3.72%. These results indicate that the use of compost and compost combined with PGPR significantly enhances grain N content, contributing positively to plant productivity in both growing seasons.

The findings from the research experiment reveal noteworthy differences in grain P content (%) across various treatments during the 2022 and 2023 growing seasons (Figure 6B). In the 50% NPK treatment, the control group exhibited grain P levels of 0.17% in 2022, which increased to 0.21% in 2023. The PGPR treatment showed higher P content at 0.22% in 2022, rising to 0.25% in 2023. The compost treatment significantly enhanced grain P, recording values of 0.34% in 2022 and increasing to 0.38% in 2023, while the compost + PGPR combination yielded the highest levels, increasing from 0.41% to 0.44%. For the 75% NPK treatment, the control group had grain P levels of 0.23% in 2022, increasing to 0.27% in 2023. The PGPR treatment demonstrated a rise from 0.31% to 0.34%, while compost showed an increase from 0.39% to 0.42%. The compost + PGPR treatment again had the highest P content, rising from 0.42% to 0.45%. In the 100% NPK treatment, the control group recorded grain P levels of 0.24% in 2022 and increased to 0.29% in 2023. The PGPR treatment levels were 0.33% in 2022 and increased to 0.36% in 2023, while the compost treatment increased from 0.39% to 0.45%. Notably, the compost + PGPR combination achieved the highest grain P content, rising from 0.45% to 0.47%. These results underscore the effectiveness of compost and compost combined with PGPR in enhancing grain P content, thereby positively influencing plant productivity across both growing seasons.

The results of the research experiment on grain K content (%) demonstrate substantial increases across various treatments during the 2022 and 2023 growing seasons (Figure 6C). In the 50% NPK treatment, the control group recorded grain K levels of 0.70% in 2022, which increased to 0.79% in 2023. The PGPR treatment showed higher levels, rising from 0.80% in 2022 to 0.84% in 2023. The compost treatment also exhibited significant growth, with grain K content increasing from 0.98% to 1.10%. The compost + PGPR combination yielded the highest K levels, increasing from 1.16% in 2022 to 1.19% in 2023. In the 75% NPK treatment, the control group had grain K levels of 0.83% in 2022, increasing slightly to 0.85% in 2023. The PGPR treatment rose from 0.87% to 0.91%, while the compost treatment saw an increase from 1.03% to 1.14%. The compost + PGPR treatment continued to demonstrate the highest levels, increasing from 1.19% to 1.23%. In the 100% NPK treatment, the control group recorded grain K levels of 0.89% in 2022, which rose to 0.92% in 2023. The PGPR treatment showed an increase from 0.98% to 1.01%, while compost levels increased from 1.15% to 1.18%. Notably, the compost + PGPR combination achieved the highest K content, increasing from 1.22% to 1.26%. These results indicate that the application of compost and compost in conjunction with PGPR significantly enhances grain K content, thereby positively influencing plant productivity throughout the two growing seasons.

The data from the research experiment highlight the variations in straw N content (%) across different treatments during the 2022 and 2023 growing seasons (Figure 6D). In the 50% NPK treatment, the control group had straw N levels of 0.71% in 2022, which increased to 0.76% in 2023. The PGPR treatment showed slightly higher levels at 0.79% for both years. In contrast, the compost treatment recorded levels of 1.05% in 2022 and increased to 1.13% in 2023, while the compost + PGPR treatment yielded the highest straw N content, rising from 1.14% to 1.17%. The 75% NPK treatment revealed similar trends, with control levels at 0.83% in 2022 and 0.84% in 2023, PGPR levels at 0.88% and 0.89%, compost showing 1.14% and 1.17%, and compost + PGPR at 1.20% and 1.25%, respectively. In the 100% NPK treatment, the control group recorded straw N contents of 0.90% in 2022 and increased to 0.93% in 2023. The PGPR treatment had levels of 0.97% in 2022 and rose to 1.02% in 2023, while compost treatments increased from 1.18% to 1.22%. The compost + PGPR combination again achieved the highest straw N content, increasing from 1.25% to 1.28%. These findings indicate that the application of compost and compost combined with PGPR significantly enhances straw N content, thereby contributing positively to overall plant productivity in both growing seasons.

The research experiment results on straw P content (%) reveal minimal yet consistent differences across various treatments during the 2022 and 2023 growing seasons (Figure 6E). In the 50% NPK treatment, both the control and PGPR groups maintained stable straw P levels at 0.03% for both years. The compost treatment also showed no change, remaining at 0.04%, while the compost + PGPR treatment recorded a slight increase from 0.05% in both years. In the 75% NPK treatment, the control group continued to show no variation, remaining at 0.03%, while the PGPR treatment slightly improved from 0.04% in 2022 to 0.04% in 2023. The compost treatment remained consistent at 0.04%, but the compost + PGPR treatment increased to 0.05% in both years. In the 100% NPK treatment, the control and PGPR groups recorded stable straw P levels at 0.04%. The compost treatment showed a slight increase from 0.05% in 2022 to 0.06% in 2023, while the compost + PGPR combination maintained the highest level, remaining at 0.06% throughout. Overall, while the increases in straw P content were relatively small, the data indicate that the application of compost and compost combined with PGPR can enhance straw P levels, suggesting their potential role in improving nutrient availability in plant systems across the two growing seasons.

The research experiment findings on straw K content (%) reveal significant improvements across various treatments during the 2022 and 2023 growing seasons (Figure 6F). In the 50% NPK treatment, the control group had straw K levels of 1.96% in 2022, increasing to 2.01% in 2023. The PGPR treatment also showed an increase, from 2.02% to 2.04%. The compost treatment exhibited a notable rise from 2.53% to 2.76%, while the compost + PGPR combination achieved the highest K levels, increasing from 2.84% in 2022 to 3.02% in 2023. In the 75% NPK treatment, the control group recorded levels of 2.04% in 2022, which increased to 2.09% in 2023. The PGPR treatment increased from 2.21% to 2.25%, and the compost treatment rose from 2.63% to 2.81%. The compost + PGPR treatment again demonstrated superior results, increasing from 2.91% to 3.18%. For the 100% NPK treatment, the control group showed an increase from 2.15% in 2022 to 2.24% in 2023, while the PGPR treatment rose from 2.26% to 2.33%. The compost treatment levels increased from 2.81% to 2.97%, with the compost + PGPR combination yielding the highest straw K content, rising from 3.22% to 3.38%. These results indicate that applying compost and compost in conjunction with PGPR significantly enhances straw K content, suggesting a positive impact on plant productivity across both growing seasons.

### 2.7. Response of Grain and Straw Yields of Wheat to Applied Treatments

**Figure 7:**
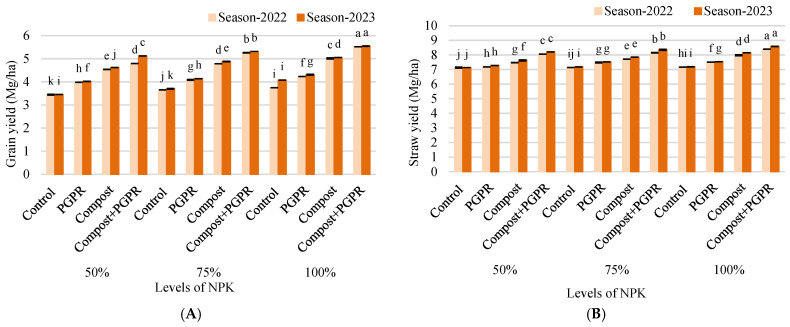
Alteration in (**A**) grain yield and (**B**) straw yield after treating wheat plants (*Triticumae stivum* L.) grown in sand soil and irrigated with low-quality water with compost, plant growth-promoting rhizobacteria (PGPR) and their combination under three levels of recommended NPK (50, 75, and 100%) during two seasons (2022 and 2023). Different letters above bars of the same season are significant at the level of *p* ≤ 0.05 according to the LSD test.

The findings from the research experiment on grain yield (Mg/ha) demonstrate significant improvements across various treatments during the 2022 and 2023 growing seasons (Figure 7A). In the 50% NPK treatment, the control group yielded 3.4 Mg/ha in 2022, increasing slightly to 3.5 Mg/ha in 2023. The PGPR treatment yielded 4.0 Mg/ha in both years, while the compost treatment showed an increase from 4.5 Mg/ha to 4.6 Mg/ha. The compost + PGPR combination produced the highest yield, rising from 4.8 Mg/ha in 2022 to 5.1 Mg/ha in 2023. In the 75% NPK treatment, the control group maintained a yield of 3.7 Mg/ha across both years. The PGPR treatment also remained consistent at 4.1 Mg/ha. The compost treatment yielded 4.8 Mg/ha in 2022 and increased to 4.9 Mg/ha in 2023, while the compost + PGPR combination achieved a yield of 5.3 Mg/ha, stable across both years. In the 100% NPK treatment, the control group improved from 3.8 Mg/ha in 2022 to 4.1 Mg/ha in 2023. The PGPR treatment showed a slight increase from 4.2 Mg/ha to 4.3 Mg/ha, and the compost treatment rose from 5.0 Mg/ha to 5.1 Mg/ha. The compost + PGPR combination again produced the highest yield, maintaining a yield of 5.5 Mg/ha throughout both seasons. These results highlight the effectiveness of compost and its combination with PGPR in enhancing grain yield, underscoring their positive impact on plant productivity during both growing seasons.

The results of the research experiment on straw yield (Mg/ha) reveal significant increases across different treatments during the 2022 and 2023 growing seasons (Figure 7B). In the 50% NPK treatment, the control group maintained a constant straw yield of 7.1 Mg/ha in both years. The PGPR treatment showed a slight increase from 7.2 Mg/ha in 2022 to 7.3 Mg/ha in 2023. The compost treatment yielded 7.5 Mg/ha in 2022 and increased to 7.6 Mg/ha in 2023, while the compost + PGPR combination produced the highest yield, rising from 8.1 Mg/ha to 8.2 Mg/ha. In the 75% NPK treatment, the control group slightly improved from 7.1 Mg/ha in 2022 to 7.2 Mg/ha in 2023. The PGPR treatment yielded 7.5 Mg/ha in both years. The compost treatment increased from 7.7 Mg/ha to 7.8 Mg/ha, while the compost + PGPR treatment demonstrated a significant rise from 8.2 Mg/ha to 8.4 Mg/ha. For the 100% NPK treatment, the control group maintained a straw yield of 7.2 Mg/ha across both growing seasons. The PGPR treatment remained consistent at 7.5 Mg/ha, while the compost treatment improved from 8.0 Mg/ha to 8.2 Mg/ha. The compost + PGPR combination yielded the highest results, increasing from 8.4 Mg/ha in 2022 to 8.6 Mg/ha in 2023. These findings indicate that the use of compost and its combination with PGPR significantly enhances straw yield, suggesting their positive influence on overall plant productivity across both growing seasons.

## 3. Discussion

The impact of compost, PGPR, and their combination on soil pH, EC_e_, and SAR across two growing seasons indicates the potential of these treatments in managing soil chemical properties under stressful conditions such as low-quality irrigation. The observed stability in soil pH across treatments, with only slight decreases over time, suggests that both compost and PGPR exert a buffering effect on soil pH, which is particularly relevant for sandy soils irrigated with low-quality water. A study on effects of living mulch using legumes on soil pH revealed a slight decrease in soil pH compared to hoeing technique for controlling weeds [11]. Compost likely contributes to this stabilization through the release of organic acids as it decomposes, which can neutralize excess alkalinity, while PGPR produces organic acids such as acetic and citric acids that further enhance pH balance [21]. These acids not only aid in moderating pH but also increase nutrient solubility, making nutrients more available for plant uptake. This is consistent with findings by [22], who noted that PGPR and compost applications helped maintain soil pH in an optimal range for plant growth. Similar decreases in soil pH were noticed when sunflower plants (*Helianthus annuus* L., cv. Sakha 53) were inoculated with PGPR compared to control under cadmium stress conditions [23].

The significant reductions in soil EC_e_ across all treatments, particularly with the compost–PGPR combination, indicate improved salinity management. Compost contributes by improving soil structure, which enhances water infiltration and leaching of salts beyond the root zone [20]. Yet, the application of living mulch in giant reed plantation (*Arundo donax* L.) slightly increased the soil EC_e_ [11]. PGPR, on the other hand, enhances root growth and biomass, further promoting salt leaching by increasing root zone penetration [23]. Similarly, usage of PGPR in sunflower plantations grown under cadmium stress resulted in a decrease in soil EC_e_ [23]. This combined effect was also observed where compost improved soil porosity and drainage, allowing salts to be effectively flushed from the root zone [24,25,26,27,28,29].

The decline in SAR is particularly noteworthy, as it suggests that the compost and PGPR combination mitigates soil sodicity. The organic matter from compost likely facilitates cation exchange, replacing sodium ions on soil particles with calcium and magnesium ions, thereby reducing SAR [9]. PGPR may also contribute by promoting root exudates that bind sodium, effectively reducing its mobility and availability for adsorption [8]. These combined mechanisms align with the findings by [30], who observed similar SAR reductions in saline soils treated with organic amendments and microbial inoculants.

The increases in soil N, P, and K content, particularly under compost–PGPR treatments, indicate that these amendments enhance nutrient availability and retention. Compost provides a slow-release source of N, P, and K as it decomposes, while PGPR can fix atmospheric nitrogen and solubilize phosphate, thereby directly increasing soil nutrient levels [31]. Furthermore, PGPRs produce enzymes that convert organic forms of P into plant-available forms, a process critical in nutrient-deficient sandy soils. This nutrient enhancement is supported by [12], who found that PGPR increased soil nutrient content, thereby improving nutrient uptake by plants in challenging soil conditions. The compost–PGPR combination yielded the highest NPK levels, suggesting a synergistic effect where compost not only contributes nutrients but also enhances the soil microbiome, providing a conducive environment for PGPR to thrive. This is corroborated by the findings of [16], who observed that organic amendments combined with microbial inoculants significantly improved soil nutrient profiles due to enhanced microbial activity and nutrient cycling. Moreover, the structural improvement provided by compost aids in retaining these nutrients within the soil, reducing nutrient leaching—a common issue in sandy soils.

The substantial increase in total bacterial and fungal counts under compost–PGPR treatments reflects the capacity of these amendments to boost microbial activity. Compost serves as a carbon source that fuels microbial growth, while PGPR enhances root exudation, providing additional nutrients for soil microbes. The presence of compost likely provides a habitat for microbial communities, shielding them from environmental stressors such as soil salinity and drought [12]. This increase in microbial biomass is essential for nutrient cycling and organic matter decomposition, which in turn improves soil fertility [30]. The compost–PGPR combination showed the highest microbial counts, likely due to the improved soil structure and enhanced root–microbe interactions facilitated by compost. The authors of [32] reported similar findings, where microbial counts were elevated by combined organic and microbial treatments, thus creating a positive feedback loop that supports nutrient availability. Enhanced microbial activity in this context not only boosts soil fertility but also contributes to disease suppression by outcompeting pathogenic microbes [33].

The increase in urease and phosphatase activities under compost–PGPR treatments suggests an improvement in nitrogen and phosphorus cycling within the soil. Urease catalyzes the hydrolysis of urea into ammonia, an essential process for nitrogen availability, while phosphatase facilitates the conversion of organic phosphorus into inorganic forms usable by plants. The presence of compost likely contributes to an increase in soil organic matter, which in turn boosts urease activity as microbial populations flourish. PGPR, known for producing extracellular enzymes, may further enhance these processes by supplying additional urease and phosphatase directly within the root zone [33]. The higher enzyme activities seen in this study align with the findings of [34], who noted that soil amendments combining organic matter and microbial inoculants significantly boosted soil enzyme activities, thus improving nutrient cycling and availability in degraded soils. The phosphatase activity observed is particularly beneficial in sandy soils, which often lack phosphorus due to rapid leaching [32]. This indicates that compost–PGPR treatments can potentially compensate for nutrient losses, making them suitable for sandy soil applications where nutrient retention is a concern.

The elevated chlorophyll content under compost–PGPR treatments reflects improved photosynthetic efficiency, likely driven by increased nutrient availability. Enhanced nitrogen and phosphorus levels are critical for chlorophyll synthesis, and the presence of these nutrients supports robust plant growth and productivity. This outcome is supported by [35], who also found that compost and microbial inoculants increased chlorophyll content by improving nitrogen availability, thereby enhancing photosynthesis.

On the other hand, proline accumulation, often a stress marker, was lower in compost and compost–PGPR treatments, suggesting reduced plant stress under these conditions. Proline functions as an osmoprotectant under drought and salinity stress, so lower proline levels imply better stress management by the plants [36]. This may be due to improved soil water retention and reduced salinity, which alleviates stress and minimizes the need for proline synthesis. The authors of [37] similarly reported reduced proline levels in compost-treated plants, attributing this to the moisture-retention capacity of organic amendments, which mitigates osmotic stress.

The yield enhancements observed in both grain and straw under compost–PGPR treatments highlight the effectiveness of these amendments in improving plant productivity in sandy soils. Compost contributes directly to biomass by supplying essential nutrients and improving soil water-holding capacity, while PGPR enhances nutrient uptake and promotes root growth, which is vital for plants grown under water-limited conditions [23]. The highest yield increases in the compost–PGPR treatment suggest a synergistic interaction where compost creates a conducive environment for PGPR, which in turn enhances nutrient uptake and growth.

The positive yield response to compost and PGPR aligns with findings by [12,38], who reported similar yield improvements in cereal crops under organic amendment and microbial inoculant treatments. These amendments appear to work together to improve both root and shoot biomass, leading to increased grain and straw yields. Moreover, the stability of yield gains across both growing seasons suggests that these treatments provide sustainable productivity improvements in challenging soil conditions, consistent with findings by [17] on the long-term benefits of organic amendments for yield stability under abiotic stress.

The limitations of this study lie primarily in its short-term assessment of compost and PGPR effects in sandy soils, as well as in the restricted environmental conditions examined. Short-term studies may not capture the cumulative benefits of organic amendments like compost, which typically show more pronounced impacts over several years [39]. Seasonal variations and climatic conditions can influence microbial dynamics, nutrient cycling, and organic matter decomposition, all of which affect the long-term efficacy of compost and PGPR in enhancing soil health [16]. Extending this research across multiple growing seasons and varying environmental conditions would provide insights into the durability of these treatments under diverse scenarios.

Additionally, while this study evaluates reduced levels of NPK, it does not fully address other stress factors like drought and high salinity, which are common in sandy soils of arid regions [40]. Investigating these treatments under such abiotic stresses could offer more robust recommendations for farmers in challenging climates. Furthermore, although the results indicate improvements in soil microbial activity and enzyme function, the specific microbial mechanisms, community structure, and diversity were not fully characterized. Employing advanced microbial analysis methods, such as metagenomics or soil enzyme profiling, could elucidate how these amendments support microbial-mediated nutrient cycling and soil stability [11].

Finally, as this study focuses on sandy soils in a specific geographic region, further research is needed to determine the applicability of these findings across various soil types and agroecosystems. Addressing these limitations could strengthen the understanding of compost and PGPR interactions with environmental factors, ultimately informing more precise, location-specific strategies for sustainable agriculture in fragile soils.

## 4. Materials and Methods

### 4.1. Experimental Description

Open field experiments were conducted in Baltim city, Kafr El-Sheikh Governorate, Egypt (31°35′57″ N 31°05′30″ E), aiming to investigate the response of wheat plants grown in sandy soil (Table 1) and irrigated with low-quality water (Table 2) to partial substitution of wheat’s recommended NPK (50%, 75%, and 100%) with compost, PGPR, or compost + PGPR during two growing seasons 2022–2023 and 2023–2024. Wheat (*Triticum aestivum* L. var. Misr 1) grains were kindly supplied from the Field Crop Research Institute, Agricultural Research Center, Department of Cereals, Sakha Agriculture Research Station, Kafr El-Sheikh, Egypt. The experiment was arranged in a split-block design with four replicates, where levels of NPK (50%, 75%, and 100% of the recommended NPK of wheat crop according to the guidelines of the Egyptian Ministry of Agriculture and Land Reclamation) occupied the main plots, while compost, PGPR, and compost + PGPR treatments represented the sub-main plots. A control was established where only NPK were applied. Seeding was conducted at a rate of 140 kg/ha in the mid of November of both seasons (2022–2023 and 2023–2024) in an experimental units of 10.5 m^2^ per each one.

The recommended NPK for wheat fertilization according to the recommendations of the Egyptian Ministry of Agriculture and Land Reclamation are 388 kg/ha urea (46% N), 238 kg/ha calcium superphosphate (15% P_2_O_5_), and 120 kg/ha potassium sulfate (50% K_2_O). The N fertilization was divided onto two equal doses and applied at 30 and 60 days after seed sowing (DAS). Both P and K fertilization were applied with soil tillage before seed sowing. For the 50% and 75% NPK treatments, the amounts of applied NPK were reduced but applied the same as the 100% NPK. Applied irrigation system was surface irrigation with a total of five irrigations with 4 weeks intervals as recommended by the Ministry of Agriculture and Land Reclamation, Egypt, with an average of 5900 m^3^/ha per each irrigation.

PGPR, i.e., *Azospirillum brasilense* SWERI 111 and *Azotobacter chroococcum* OR512393, were kindly provided by the Microbiology Department, Soils, Water and Environment Research Institute, Agricultural Research Center, Sakha Agriculture Research Station, Kafr El-Sheikh, Egypt. Bacteria were grown on Nutrient Broth liquid medium (NB) with typical formula (g/L); beef extract 1.0, peptone 5.0, yeast extract 2.0, and sodium chloride 3.0. The final pH was adjusted to 6.8 ± 0.2 at 28 °C. The pure isolates were grown in 500 mL flask containing 250 mL NB on a rotary shaker incubator at 28^◦^ C for 8 h daily. After 3 days of inoculations, peat-based cultures of isolates were prepared using the method described by [41]. Cell suspensions containing 10^7^ CFU/mL were used to impregnate sterilized peat at the rate of 50 mL NB per 100 g peat. Inoculated peat was well mixed and allowed to mature at room temperature for 48 h. Wheat seeds were mixed, just before sowing, with PGPR peat inoculum at a rate of 950 g/ha, after being wetted with 10% Arabic gum water solution as an adhesive material [42]. Seeds were allowed to air dry in the shad for 30 min.

The used compost in the present study, as an organic fertilizer, was kindly obtained from the Microbiology Department, Sakha Agricultural Research Station, Kafr El-Sheikh, Egypt. The compost consisted of 50% plant materials and 50% animal waste. This combination is commonly used in composting because it balances the carbon-to-nitrogen ratio, which is crucial for the decomposition process. It was applied at a rate of 20 ton/ha during soil tillage ensuring the homogenous distribution. The physical and chemical properties of the compost are presented in the Table 3.

### 4.2. Sampling and Measurements

#### 4.2.1. Soil-Related Traits

At the end of the experiment, soil samples were collected from the upper soil horizon (0–20 cm) in triplicate per each experimental plot. Collected soil samples were air-dried, crushed, sieved through a 2 mm sieve, and subjected to chemical analysis, as described by [43]. For soil biochemical analysis, other soil samples were gathered also in triplicates, sieved through an 8 mm sieve after removal of gravels, stones, and plant remains, and kept in polyethylene bags at −20 °C for further analysis.

Soil pH was determined in soil-water suspension (1:2.5) using pH meter (Jenway 3510, Cole-Parmer, Westwood Ave, Long Branch, USA), while soil electrical conductivity (EC_e_) was measured in soil paste extract using EC meter (Jenway 4310, USA). The soil sodium adsorption ratio (SAR) is an important parameter for assessing soil sodicity, which can affect soil structure, permeability, and plant growth. The SAR value is calculated using the concentrations of sodium (Na^+^), calcium (Ca^2+^), and magnesium (Mg^2+^) ions in the soil paste extract (meq/L) using the following formula [43]:

$$SAR=\frac{\left[{Na}^{+}\right]}{\sqrt[{2}]{\frac{\left[{Ca}^{2+}\right]\;+\;[{Mg}^{2+}]}{2}}}$$

Soil organic matter (SOM) content was determined by the Walkley–Black chromic acid wet oxidation method using fine powder of air-dried soil samples (<0.25 mm) [44].

For determination of available soil NPK, 1 M potassium chloride, 0.5 M sodium bicarbonate, and 1 M ammonium acetate (pH 7) solutions were applied to extract N, P, and K, respectively. The available N was determined according to the Kjeldahl method [44], while available P was quantified spectrophotometrically according to the ascorbic acid method [45] using the Amersham Biosciences Ultrospec 2100 Pro UV/Visible (Holliston, MA, USA). The extracted K according to [44] was determined using the flame photometer (Sherwood Model 410; Incheon, Republic of Korea), as described by [46].

Total bacterial count in the soil samples was determined according to [47] at 80 days of seed sowing. The numbers of fungi in the soil are usually determined by the plate method used by [48]. Phosphate-solubilizing bacteria count was measured by determining the number of colonies with clear halos (a sign of solubilization) [49]. The number of viable cells was calculated using the following formula: number of cells per mL (CFU/g soil) = (number of colonies) × (dilution factor).

Eighty days after seed sowing, soil samples were collected to determine soil enzyme activities. Phosphatase activity was assessed using the protocol outlined by [50]. Soil samples were incubated with p-nitrophenyl phosphate, and the resulting color intensity was measured colorimetrically at 440 nm. Urease activity was determined following the method of [26], where soil samples were incubated with a 2% aqueous urea solution, and residual urea was measured colorimetrically at 527 nm.

#### 4.2.2. Plant-Related Traits

Relative chlorophyll content (SPAD) was quantified 80 days after seed sowing in 6 repeats per plot using youngest fully expanded leaves by the Minolta chlorophyll meter SPAD-502.

Proline serves as an essential osmolyte and osmoprotective compound. The leaf proline content was determined using the method developed by [27]. In brief, 0.5 g of the uppermost fully expanded leaves, collected 80 days after seed sowing, were ground with 3% sulfuric acid and centrifuged at 12,000× *g* for 5 min. The resulting solution was quantified using the ninhydrin reagent. The supernatant was then homogenized with toluene, and the absorbance was measured at 520 nm using a UV-160A spectrophotometer (Shimadzu, Kyoto, Japan).

At harvest, 10 g of grains from each treatment were selected, air-dried, crushed, and prepared for laboratory analysis to determine the grain N, P, and K content. The nitrogen content was measured using the micro Kjeldahl method [28], phosphorus content was analyzed using a spectrophotometer, and potassium content was determined with a flame photometer, following the procedure described by [44]. Zinc content (mg/kg) in the grains and straw of wheat plants was assessed in finely ground samples after digestion with a HNO_3_:HClO_4_ (3:1 *v*/*v*) mixture, using an Atomic Absorption Spectrophotometer (AAS, Perkin Elmer 3300) with a detection limit of 100 ppb, as described by [44]. The grain and straw yields were recorded after the harvest of the experimental plots.

### 4.3. Statistical Analysis

The obtained collected data were subjected to statistical analysis, using the analysis of variance (ANOVA). The LSD range tests were used to compare between the means [29].

## 5. Conclusions

The findings of this study indicate that the combined application of compost and PGPR offers substantial benefits for wheat cultivation in sandy soils, especially under conditions of varying NPK fertilization. The integrated treatment consistently improved soil health indicators, including reductions in EC and SAR, and increased SOM, supporting a more conducive environment for wheat growth. Enhanced microbial activity and enzyme functions from the combined treatment facilitated better nutrient cycling and availability, crucial for sustaining soil fertility. Importantly, the increase in chlorophyll content and decrease in proline levels suggest that compost and PGPR alleviate plant stress, further promoting higher yields. Overall, the compost and PGPR combination emerges as a promising strategy to enhance productivity sustainably, particularly in nutrient-limited and low-quality water-irrigated soils. Further research is warranted to explore the long-term implications of these amendments on soil health and crop resilience under diverse environmental conditions.

## Figures and Tables

**Table 1:** Features of the experimental soil during the 2022 and 2023 seasons.

Parameter	2022	2023
pH (1:2.5 soil: distilled water suspension)	7.87 ± 0.01	7.86 ± 0.01
EC ^¥^ (Soil paste extract; dS/m)	3.36 ± 0.03	2.84 ± 0.02
Soluble ions (meq/L)		
Na^+^	19.86 ± 0.95	16.78 ± 0.84
K^+^	0.27 ± 0.01	0.23 ± 0.01
Ca^2+^	7.02 ± 0.51	5.94 ± 0.42
Mg^2+^	6.65 ± 0.33	5.62 ± 0.25
CO_3_^2−^	nd ^†^	nd
HCO_3_^−^	10.24 ± 0.72	9.72 ± 0.61
Cl^−^	14.26 ± 0.88	12.05 ± 0.83
SO_4_^2−^	9.30 ± 0.31	6.79 ± 0.22
SAR (Sodium adsorption ratio)	7.59 ± 0.12	6.98 ± 0.15
Exchangeable sodium percentage (%)	6.53 ± 0.11	6.44 ± 0.13
Available macronutrients (mg/kg)		
N	22.75 ± 1.21	23.62 ± 1.24
P	5.22 ± 0.21	5.35 ± 0.19
K	197 ± 21	198 ± 25
Bulk density (kg/m^3^)	1.23 ± 0.01	1.22 ± 0.02
Total porosity (%)	53.58 ± 3.41	53.96 ± 3.21
Organic matter (%)	0.53 ± 0.01	0.54 ± 0.01
CaCO_3_ (%)	1.63 ± 0.02	1.65 ± 0.02
Field capacity (%)	13.32 ± 0.32	13.22 ± 0.29
Wilting point (%)	5.25 ± 0.22	5.24 ± 0.19
Cation exchange capacity (cmolc/kg)	0.86 ± 0.01	0.85 ± 0.01
Particle size distribution (%)		
Coarse sand	37.56 ± 1.11	37.71 ± 1.01
Fine sand	54.66 ± 2.01	53.75 ± 1.99
Silt	3.28 ± 0.01	3.94 ± 0.01
Clay	4.50 ± 0.03	4.60 ± 0.04
Texture class	Sandy	Sandy

^¥^ electrical conductivity; ^†^ not detected.

**Table 2:** Characterization of the irrigation water used in 2022 and 2023 seasons.

Parameter	2022	2023
pH	7.83 ± 0.02	7.77 ± 0.01
EC ^¥^ (dS/m)	1.12 ± 0.03	1.09 ± 0.01
Soluble ions (meq/L)		
Ca^2+^	2.34 ± 0.11	2.28 ± 0.12
Mg^2+^	1.34 ± 0.06	1.31 ± 0.04
Na^+^	7.60 ± 0.07	7.40 ± 0.05
K^+^	0.09 ± 0.001	0.08 ± 0.002
CO_3_^2−^	nd ^†^	nd
HCO_3_^−^	3.15 ± 0.03	3.63 ± 0.02
Cl^−^	5.31 ± 0.06	5.17 ± 0.08
SO_4_^2−^	2.91 ± 0.02	2.27 ± 0.04
SAR (Sodium adsorption ratio)	5.59 ± 0.06	5.52 ± 0.05

^¥^ electrical conductivity; ^†^ not detected.

**Table 3:** Properties of the compost applied in the experiment in 2022 and 2023 seasons.

Parameter	2022	2023
pH (1:10 compost: distilled water suspension)	6.87 ± 0.01	6.69 ± 0.01
EC ^¥^ (1:10 compost: distilled water extract; dS/m)	4.61 ± 0.05	4.57 ± 0.04
Organic matter (%)	37.84 ± 2.22	37.82 ± 1.98
N (%)	1.44 ± 0.03	1.42 ± 0.04
C (%)	33.14 ± 0.92	32.65 ± 0.88
C:N	23.01 ± 1.01	22.99 ± 0.99
P (%)	0.78 ± 0.02	0.77 ± 0.03
K (%)	1.31 ± 0.13	1.16 ± 0.11
Manganese (mg/kg)	341 ± 25	339 ± 26
Iron (mg/kg)	329 ± 32	331 ± 27
Zinc (mg/kg)	76 ± 11	74 ± 10

^¥^ electrical conductivity.

## Data Availability

Data will be made available upon the request.
